# *microRNA*-*21* expressions impact on liver fibrosis in biliary atresia patients

**DOI:** 10.1186/s13104-019-4227-y

**Published:** 2019-03-29

**Authors:** Akhmad Makhmudi, Alvin Santoso Kalim, Gunadi

**Affiliations:** Pediatric Surgery Division, Department of Surgery, Faculty of Medicine, Public Health and Nursing, Universitas Gadjah Mada/Dr. Sardjito Hospital, Jl. Kesehatan No. 1, Yogyakarta, 55281 Indonesia

**Keywords:** Biliary atresia, Biliary cirrhosis, Liver fibrogenesis, *miRNA*-*21*, *PTEN*, qPCR

## Abstract

**Objective:**

Biliary atresia (BA) is the most common cause of neonatal jaundice, characterized by progressive and rapid liver fibrosis. Recent studies have shown that microRNAs (miRNAs) contribute to the liver fibrogenesis. We investigated the *miRNA*-*21* impact in liver fibrogenesis in Indonesian BA patients.

**Results:**

There were 5, 4, and 7 BA patients with type 2A, 2B, and 3, respectively. Quantitative real-time polymerase chain reaction (qPCR) showed that the *miRNA*-*21* expression was significantly increased (18-fold) in BA patients compared to controls (− 4.4 ± 4.0 vs. − 0.2 ± 4.8; *p *= 0.041). Furthermore, the *phosphatase and tensin homolog deleted on chromosome ten (PTEN)* expression was significantly down-regulated (3.1-fold) in BA group compared to control group (0.2 ± 1.4 vs. − 1.4 ± 1.7; *p *= 0.036). The *α*-*smooth muscle actin* (*α*-*SMA*) expression was not statistically significantly different between groups (13.7 ± 3.8 vs. 15.0 ± 4.8; *p *= 0.87). Interestingly, the *miRNA*-*21* expression was significantly lower (25-fold) in cirrhosis than non-cirrhosis BA patients (− 0.8 ± 2.2 vs. − 5.3 ± 3.9; *p *= 0.004). In conclusions, our study provides support for the association between *miRNA*-*21* expression and liver cirrhosis in BA patients. Further study with a larger sample size of patients is important to confirm our results.

**Electronic supplementary material:**

The online version of this article (10.1186/s13104-019-4227-y) contains supplementary material, which is available to authorized users.

## Introduction

Biliary atresia (BA) is the most common cause of neonatal jaundice, characterized by progressive and rapid liver fibrosis and often resulting in the cause of death of children under 2 years of age if the Kasai procedure is not conducted at an early age [1, 2]. BA incidence differs among ethnic populations, involving 1:7000 live births in Indonesia [3–5].

There are several hypotheses proposed for the development of BA, including epigenetic factors such as microRNAs (miRNAs) [6]. One of the predominant hypotheses is the increased *miRNA*-*21* expression in the liver of BA patients [7]. The increased *miRNA*-*21* will inhibit the *phosphatase and tensin homolog deleted on chromosome ten* (*PTEN*) through its 3′-untranslated region (UTR) and enhance the *α*-*SMA* expressions [7, 8]. Furthermore, it has been shown that the *miRNA* expression might be different among ethnic groups [9, 10]. Nevertheless, the allele frequencies of some common variants are different within Asia [11] and the risk allele frequency of *SEMA3* rs11766001 polymorphism had different impacts on the development of Hirschsprung disease depending on the ethnic background [12]. Therefore, we aimed to determine the effect of *miRNA*-*21* expression in liver fibrogenesis in BA patients in an Indonesian population.

## Main text

### Methods

#### Patients

This study was conducted from June 2015 to October 2017 at Dr. Sardjito Hospital, Yogyakarta, Indonesia [13, 14]. The inclusion criteria were infants with a diagnosis of BA by clinical features, laboratory findings, abdominal ultrasound, intraoperative cholangiography, magnetic resonance cholangiopancreatography, and liver biopsy, whereas the exclusion criteria were total RNA with low quality. For post-operative laboratory findings, we took the data at 1 week after Kasai surgery.

Parents of the BA patients and controls signed written informed consent before participating in this study. An approval was given by the Institutional Review Board of the Faculty of Medicine, Public Health and Nursing, Universitas Gadjah Mada/Dr. Sardjito Hospital for this study (KE/FK/528/EC/2015).

#### Liver cirrhosis

We classified the histopathology of liver biopsy in BA patients according to the Laennec system as follows: 0, no definite fibrosis; 1, minimal fibrosis (no septa or rare thin septum; may have portal expansion or mild sinusoidal fibrosis); 2, mild fibrosis (occasional thin septa); 3, moderate fibrosis (moderate thin septa; up to incomplete cirrhosis); and 4, cirrhosis [15]. Next, we allocated the grading of 0 to 3 for fibrosis into the non-cirrhosis group for further analysis.

#### Total RNA isolation and quantitative real-time polymerase chain reaction

The miRCURY™ RNA Isolation Kit-Tissue (Exiqon A/S, Denmark) was used to extract the total RNA from liver tissue. The quantitative real-time polymerase chain reaction (qPCR) was performed to determine the expression of *miRNA*-*21, PTEN*, and *α*-*SMA* using the BioRad CFX Real-Time PCR System (California, USA), the Universal cDNA Synthesis Kit II (Exiqon A/S, Denmark), ExiLENT SYBR^®^ Green Master Mix Kit (Exiqon A/S, Denmark), and miRCURY™ LNA™ Universal RT microRNA PCR System (Exiqon A/S, Denmark). *U6 snRNA* was used as a control for analysis of *miRNA*-*21* expression, whereas *glyceraldehyde*-*3*-*phosphate dehydrogenase* (*GAPDH*) served as a reference gene for analysis of *PTEN* and *α*-*SMA* expression.

The primers of *hsa*-*miRNA*-*21 and U6 snRNA* were 5′-GTCGTATCCAGTGCGTGTCGTGGAGTCGGCAATTGCACTGGATACGACACAGCCCA-3′ (RT), 5′-GCGGCAACACCAGTCGATG-3′ (forward), and 5-’TGCGTGTCGTGGAGTC-3′ (reverse); and 5′-AAAATATGGAACGCTTCACGAATTTG-3′ (RT), 5′-GCTTCGGCAGCACATATACTAAAAT-3′ (forward) and 5′-CGCTTCACGAATTTGCGTGTCAT-3′ (reverse), respectively [16], while the primer sequence for *PTEN*, *α*-*SMA*, and *GAPDH* were 5′-ACCCCTTCATTGACCTCAACTA-3′ (forward) and 5′-TCTCGCTCCTGGAAGATGGTGA-3′ (reverse); 5′-GACAATGGCTCTGGGCTCTGTAA-3′ (forward) and 5′-CTGTGCTTCGTCACCCACGTA-3′ (reverse); and 5′-GCACCGTCAAGGCTGAGAAC-3′ (forward) and 5′-TGGTGAAGACGCCAGTGGA-3′ (reverse), respectively [7].

We utilized the Livak (2^−ΔΔC^_T_) method to determine the *miRNA*-*21*, *PTEN* and *α*-*SMA* expressions [17].

#### Statistical analysis

The *miRNA*-*21*, *U6 snRNA*, *PTEN*, *α*-*SMA*, and *GAPDH* expression were determined as mean values ± SD and Mann–Whitney U test was used to search for statistical differences between groups. A *p* value < 0.05 was considered statistically significant.

### Results

#### Baseline characteristics

We ascertained 17 liver specimens from BA patients and seven liver samples from abdominal trauma patients as controls. The controls were one male and six females with their mean age during laparotomy of 5.3 ± 4.8 years. We excluded one BA patient because of low quality of total RNA, thus, we further investigated 16 BA patients and seven controls. All *miRNA*-*21* and *PTEN/α*-*SMA* expression levels in the subgroups refer to the same set of liver biopsies that were obtained during the Kasai procedure.

There were 16 BA patients, of whom seven males and nine females, and most patients were type 3 BA. The mean age at Kasai procedure was 106.8 ± 54.2 days, with the survival rate of 43.8% (Table 1).

**Table 1:** Characteristics of BA patients following Kasai procedure in Dr. Sardjito Hospital, Indonesia

Characteristics	N (%); mean ± SD
Sex	
Male	7 (43.8)
Female	9 (56.2)
BA type	
1	0
2A	5 (31.2)
2B	4 (25)
3	7 (43.8)
Pre-operative laboratory findings	
Total bilirubin (mg/dL)	10.7 ± 4.2
Direct bilirubin (mg/dL)	8.1 ± 3.2
Alanine aminotransferase (ALT) (U/L)	148.3 ± 72.6
Aspartate aminotransferase (AST) (U/L)	282.3 ± 222.3
Alkaline phosphatase (ALP) (U/L)	508.7 ± 181.3
Gamma glutamyl transferase (GGT) (U/L)	471.2 ± 324.6
Age at Kasai procedure (days)	106.8 ± 54.2
Post-operative laboratory findings	
Total bilirubin (mg/dL)	9.2 ± 5.0
Direct bilirubin (mg/dL)	7.0 ± 3.3
Alanine aminotransferase (ALT) (U/L)	170.0 ± 148.6
Aspartate aminotransferase (AST) (U/L)	238.2 ± 363.0
Alkaline phosphatase (ALP) (U/L)	243.8 ± 120.6
Gamma glutamyl transferase (GGT) (U/L)	492.7 ± 516.8
Post-operative clinical findings	
Ascites	7 (43.8)
Cholangitis	6 (37.5)
Sepsis	10 (62.5)
Portal hypertension	6 (37.5)
Esophageal varices	5 (31.3)
Histopathological findings of liver biopsy	
Cirrhosis	4 (25)
Non-cirrhosis	12 (75)
Survival rate	7 (43.8)

*BA* biliary atresia

#### Association between miRNA-21, PTEN and α-SMA expressions and BA

qPCR demonstrated that the *miRNA*-*21* expression was significantly increased (18-fold) in BA patients compared to the controls (− 4.4 ± 4.0 vs. − 0.2 ± 4.8), with *p*-value of 0.041 (Table 2 and Additional file 1: Figure S1).

**Table 2:** The *miRNA*-*21*, *PTEN and α*-*SMA* expressions in the BA patients and control liver

	Biliary atresia (ΔC_T_ ± SD)	Control (ΔC_T_ ± SD)	ΔΔC_T_ (95% CI)	Fold change (2^−ΔΔC^_T_)	*p*-value
*miRNA*-*21*	− 4.4 ± 4.0	− 0.2 ± 4.8	− 4.2 [− 8.2 to − 0.2]	18	0.041*
*PTEN*	0.2 ± 1.4	− 1.4 ± 1.7	1.6 (0.2 to 3.0)	3.1	0.036*
*α*-*SMA*	13.7 ± 3.8	15.0 ± 4.8	–1.4 (− 5.3 to 2.5)	2.6	0.87

* Mann–Whitney U test is used to search for statistical differences between groups and *p *< 0.05 is considered statistically significant

Furthermore, the *PTEN* expression was down-regulated (3.1-fold) in the BA group compared to the control group (0.1 ± 1.4 vs. − 1.1 ± 1.8), and reached a significant level (*p *= 0.036). The *α*-*SMA* expression was not statistically significantly different in BA patients compared to the controls (13.7 ± 3.8 vs. 15.0 ± 4.8; *p *= 0.87) (Table 2 and Additional file 1: Figure S1).

#### Association between miRNA-21, PTEN and α-SMA expressions and outcomes of BA patients after Kasai procedure

Next, we compared the expression of *miRNA*-*21*, *PTEN* and *α*-*SMA* between cirrhosis and non-cirrhosis BA patients. Interestingly, the *miRNA*-*21* expression was significantly lower (25-fold) in cirrhosis than non-cirrhosis BA patients (− 0.8 ± 2.2 vs. − 5.3 ± 3.9; *p *= 0.004); whereas there was no difference of *PTEN* (*p *= 0.76) and *α*-*SMA* (*p *= 0.44) expressions between the two groups (Table 3). In addition, the expressions of *miRNA*-*21*, *PTEN* and *α*-*SMA* did not correlate with the age when the Kasai procedure was performed (*p *= 0.65, 0.45, and 0.39), nor with BA patient’s survival (*p *= 0.22, 0.35, and 0.52), respectively (Table 3).

**Table 3:** Association between *miRNA*-*21, PTEN and α*-*SMA* expressions and outcomes of BA patients after Kasai procedure

	*miRNA*-*21*	*PTEN*	*α*-*SMA*
Age at Kasai operation of ≥ 90 days (n = 9)	− 3.6 ± 4.4	0.01 ± 0.9	12.7 ± 3.6
Age of Kasai operation of < 90 days (n = 7)	− 2.5 ± 3.2	0.2 ± 1.9	13.8 ± 2.5
ΔΔC_T_ (95% CI)	− 1.1 (− 4.4 to 2.2)	− 0.2 (− 1.7 to 1.3)	− 1.2 (− 3.9 to 1.6)
Fold change (2^−ΔΔC^_T_)	2.1	1.1	2.3
*p*-value	0.65	0.45	0.39
Cirrhosis (ΔC_T_ ± SD) (n = 4)	− 0.8 ± 2.2	0.4 ± 2.3	12.4 ± 1.4
Non-cirrhosis (ΔC_T_ ± SD) (n = 12)	− 5.3 ± 3.9	− 0.03 ± 0.9	13.9 ± 4.3
ΔΔC_T_ (95% CI)	4.5 (1.8–7.2)	0.4 (− 1.2 to 2.0)	− 1.5 (− 4.2 to 1.2)
Fold change (2^−ΔΔC^_T_)	25	1.3	2.8
*p*-value	0.004*	0.76	0.44
Died (ΔC_T_ ± SD) (n = 9)	− 2.1 ± 4.1	0.3 ± 1.6	13.4 ± 3.2
Survived (ΔC_T_ ± SD) (n = 7)	− 4.1 ± 3.4	− 0.2 ± 1.0	12.5 ± 3.2
ΔΔC_T_ (95% CI)	1.9 (− 1.3 to 5.2)	0.5 (− 1.0 to 2.0)	0.9 (− 1.9 to 3.6)
Fold change (2^−ΔΔC^_T_)	3.8	1.5	1.8
*p*-value	0.22	0.35	0.52

* Mann–Whitney U test is used to search for statistical differences between groups and *p *< 0.05 is considered statistically significant

### Discussion

We describe new data on the *miRNA*-*21* expression in Indonesian BA patients. We were able to find evidence of the impact of *miRNA*-*21* in the liver fibrotic process in Indonesian BA patients by showing its expression is 18-fold increase in the BA patients compared to the control livers. Interestingly, the *miRNA*-*21* expression also differed between cirrhosis and non-cirrhosis BA patients. These evidences strongly support the role of *miRNA*-*21* in the liver fibrogenesis in BA patients.

The up-regulation of *miRNA*-*21* has been hypothesized to be implicated in the pathogenesis of liver fibrotic process in BA patients through inhibition of *PTEN* via the 3′-UTR and increasing *α*-*SMA* expression [7, 8]. Furthermore, previous study also showed that *miRNA*-*21* was strongly activated in a BA animal model liver, implying its significance in cellular growth [18]. Our study provides additional support for this inverse connection between increased *miRNA*-*21* and lower *PTEN* expression and contributes new data from a population genetically different [12] from previous study (Indonesian vs. Chinese) [7]. It has been reported that some common variants might have different frequencies of their risk alleles among Asians [11]. Another novelty of our study is that we associated the *miRNA*-*21* expression with the grade of liver fibrosis (cirrhosis vs. non-cirrhosis BA patients), age at Kasai procedure (≥ 90 vs. < 90 days), and clinical outcome (died vs. survived patients). Our study also shows that the *PTEN* expression was down-regulated in our BA patients’ livers compared to the control livers further contributing support for the previous finding in a Chinese population.

It has been shown that *α*-*SMA* was highly expressed during transdifferentiation of hepatic stellate cells into myofibroblasts during liver fibrogenesis [19]. The *α*-*SMA* expression was not statistically significant difference in our BA patients compared to the controls. This lack of statistical power might relate to the small sample size, indicating that a larger sample size is important to be ascertained to confirm our findings.

miRNAs, small non-coding RNAs that deregulate expression of gene at the posttranscriptional level, are stable and easily measureable in the patients’ tissue and blood specimens, including BA patients’ livers [7, 20–22]. Although several miRNAs have been shown to have a role in the pathogenesis of BA [7, 20, 21], the BA pathogenesis and diagnostic usefulness of miRNAs remains inadequate [21]. Therefore, it is always challenging and interesting to look for which miRNAs and their targets have the strongest impact on liver fibrogenesis to determine those miRNAs as potential biomarkers and/or molecular therapy for BA patients in the future. Interestingly, recent study showed that anti-miRNA-21 reduces the liver fibrosis in hepatocytic deletion of *Pten* mice [23].

This study provides new results to extend the knowledge on mechanisms causing liver fibrogenesis in BA patients. The probability of the progression of liver fibrosis after a Kasai procedure should be described during counseling to BA parents.

### Conclusions

Our study provides support for the association between *miRNA*-*21* expression and liver cirrhosis in BA patients. Further study with a larger sample size of patients is important to confirm our results.

## Limitation

We noticed that our small sample size indicates that more patients needs to be involved to confirms and clarifies our results. Another weakness of our study was use of only a single normalizer both in the *miRNA*-*21* (i.e. *U6 snRNA*) and *PTEN/α*-*SMA* (i.e. *GAPDH*) qPCR [24]. The use of multiple normalizers would likely decrease some of the experimental noise in the qPCR data. The large SD for some data in our study might imply overlap in the C_Ts_ between the normalizer and the transcripts of interest. Therefore, further study with additional normalizers (i.e. *RNU44, RNU48*, and *miRNA*-*16* for *miRNA*-*21*, and *ACTB, YWHAZ* and *IPO8* for *PTEN*/*α*-*SMA* expressions) is necessary to overcome this issue.

## Additional file

**Additional file 1: Figure S1.** Box-plot graph of ΔC_T_ value of the *miRNA-21, PTEN* and *α-SMA* expressions in liver BA patients (white box) and controls (black box). Box-plot graph of ΔC_T_ value reveals the median values as lines across the box. Lower and upper boxes are representing the 25th percentile to the 75th percentile, while whiskers are indicating the maximum and minimum values.

## Abbreviations

*α*-*SMA*: *α*-*smooth muscle actin*
BA: biliary atresia
*GAPDH*: *glyceraldehyde*-*3*-*phosphate dehydrogenase*
*miRNA*-*21*: *microRNA*-*21*
*PTEN*: *phosphatase and tensin homolog deleted on chromosome ten*
qRT-PCR: quantitative real-time polymerase chain reaction
