# Supplementary figures and images for: microRNA-21 expressions impact on liver fibrosis in biliary atresia patients
Source: BMC Res Notes. 2019 Mar 29;12:189. doi: 10.1186/s13104-019-4227-y (PMC6441216; doi:10.1186/s13104-019-4227-y)

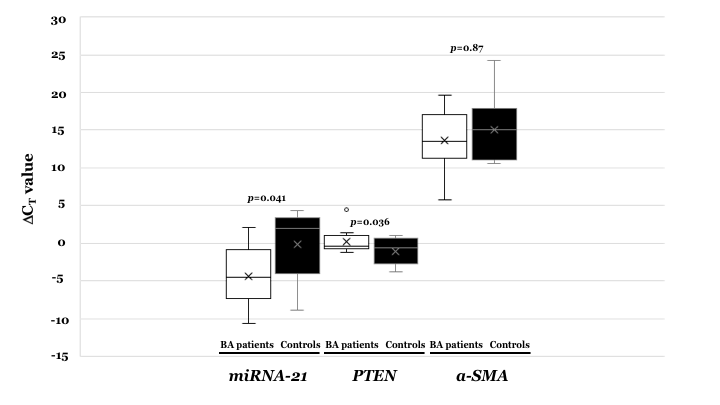

Supplement: Supplementary file 1 — Additional file 1: Figure S1. Box-plot graph of ΔCT value of the miRNA-21, PTEN and α-SMA expressions in liver BA patients (white box) and controls (black box). Box-plot graph of ΔCT value reveals the median values as lines across the box. Lower and upper boxes are representing the 25th percentile to the 75th percentile, while whiskers are indicating the maximum and minimum values. [file 13104_2019_4227_MOESM1_ESM.tiff]
